# Lichen Sclerosus—Incidence and Comorbidity: A Nationwide Swedish Register Study

**DOI:** 10.3390/jcm13102761

**Published:** 2024-05-08

**Authors:** Sandra Jerkovic Gulin, Filippa Lundin, Olle Eriksson, Oliver Seifert

**Affiliations:** 1 Department of Dermatology and Venereology, Ryhov County Hospital, Sjukhusgatan, 553 05 Jönköping, Sweden; 2 Division of Cell Biology, Department of Biomedical and Clinical Sciences, Faculty of Medicine and Health Sciences, Linköping University, 581 83 Linköping, Sweden; 3 Faculty of Medicine and Health Sciences, Linkoping University, 581 83 Linköping, Sweden; 4 Futurum-Academy for Healthcare, Region Jönköping County, 553 05 Jönköping, Sweden

**Keywords:** lichen sclerosus, vulva cancer, breast cancer, penile cancer, bladder cancer, testicular cancer, comorbidity

## Abstract

**Background**: Data on the incidence and comorbidity of Lichen sclerosus (LS), based on validated nationwide population-based registries, remains scarce. **Objective**: To explore the incidence and association of comorbidities with LS in Sweden, emphasizing its potential links to malignancies and autoimmune disorders. **Methods**: A population-based retrospective open cohort study was conducted using the National Patient Register to identify all individuals diagnosed with LS (ICD-10 code L90.0) from 1 January 2001 to 1 January 2021. The study included 154,424 LS patients and a sex and age matched control group of 463,273 individuals to assess the incidence and odds ratios for various cancers and premalignant conditions. **Results**: The incidence of LS in Sweden was 80.9 per 100,000 person per year, with higher incidence in females (114.4) than in males (47.2). LS patients showed an increased odds ratio for vulvar cancer (OR = 8.3; 95% CI = 7.5–9.0), penile cancer (OR = 8.9; 95% CI = 7.3–11.0), prostate cancer (OR = 1.2; 95% CI = 1.1–1.2), testicular cancer (OR = 1.4; 95% CI = 1.1–1.7), bladder cancer (OR = 1.1; 95% CI = 1.1–1.2), breast cancer (OR = 1.4; 95% CI = 1.3–1.4), leukoplakia of the vulva (OR = 253.5; 95% CI = 221.9–289.6), and leukoplakia of the penis (OR = 5.1; 95% CI = 4.9–5.4). **Conclusions**: This study underscores the significantly increased association of various cancers and premalignant conditions in LS patients, highlighting the critical need for efficacious treatment and diligent follow-up. The association between LS and autoimmune diseases further necessitates comprehensive investigation to understand the underlying mechanisms and clinical management implications. Future research is essential to confirm these findings and elucidate the role of LS in cancer development.

## 1. Introduction

Lichen sclerosus (LS) represents a chronic, lymphocyte-mediated inflammatory condition that primarily affects mucocutaneous regions, with a notable predilection for anogenital sites [1]. Manifesting with severe itching, pain due to erosions or fissures, and scarring, LS, if not adequately addressed, can progress to a severe stage [2,3,4]. This advanced stage is characterized by significant alterations in genital structural anatomy in both genders, encompassing ulcerative lesions with hemorrhage on the vulva, diminution of the labia minora, scarring and narrowing of the vaginal introitus, urine retention, anal stenosis, constipation, and phimosis. Consequently, individuals with LS may suffer from sexual and urinary dysfunction, necessitating reconstructive surgical interventions. The primary therapeutic approach involves the application of potent topical steroids (clobetasol ointment) to the affected regions. In male patients, circumcision may serve as a curative treatment option when topical steroids fail [1,5,6,7,8,9,10]. Delayed diagnosis or steroid resistance may compel the need for genital reconstruction to restore functionality. Moreover, neglected LS in the vulvar area can evolve into a premalignant state or progress to vulvar squamous cell carcinoma (SCC), while penile LS poses a risk for penile SCC [9,11,12,13,14,15].

The precise pathophysiological mechanisms and etiology of LS remain unclear, though hypotheses suggest autoimmune, isotraumatopic, or infectious origins. Research has identified circulating IgG autoantibodies against extracellular matrix protein 1 (ECM1) in affected individuals of both sexes [16]. Associations with human leukocyte antigen (HLA) class II have been observed in penile LS cases, whereas similar HLAs may offer protection in vulvar LS [17]. Theories regarding LS development include chronic exposure to urine, with circumcision often being curative in early stages [6,13,18,19], and occurrences of extragenital LS in areas affected by radiation therapy [20], indicating a potential predisposition following trauma [1]. Notably, a genetic component is suggested by family history reports in 12% of LS cases [17]. Comprehensive studies are imperative for elucidating LS etiology.

LS can affect individuals across all age groups, but females are generally more susceptible than males. The disease presents in two peak age groups for vulvar LS: prepubertal and postmenopausal years [2,4,6,21]. Similarly, LS in males also shows a bimodal onset pattern, affecting young boys and adult men. The scarcity of large-scale epidemiological research limits our understanding of the true incidence of LS [22,23,24]. Estimates suggest a prevalence ranging from 1:60 to 1:1000 among adults and children in the United States [25]; however, due to the potential for asymptomatic cases and the disease being under-recognized, these figures likely underestimate the true prevalence [22]. Increases in vulvar LS incidence and the associated risk of vulvar SCC have been documented, alongside a noted lifetime risk of penile SCC in affected males [12,13,14,15,24]. However, no epidemiological studies specifically addressing LS have been conducted in Sweden.

The correlation between LS and vulvar SCC is well-established [26], but research into the association of LS with other cancers remains limited. Links between LS and conditions such as thyroid disease, psoriasis [27,28], vitiligo [29,30], lichen planus [4,8,11], alopecia areata, and ulcerative colitis [21,31,32] have been explored in various studies and case reports. The co-occurrence of LS with morphea has been sporadically reported, though the nature of this relationship remains a subject of debate [33]. The prevalence of comorbid conditions in Swedish LS patients has not been thoroughly examined.

Current awareness of LS among medical professionals is inadequate, often leading to misdiagnosis, delayed treatment, and insufficient follow-up. This lack of awareness, coupled with a general reluctance to seek medical care for genital symptoms, exacerbates the risk of severe genital disease, urogenital dysfunction, and, potentially, cancer development. Enhancing our understanding of LS is thus crucial.

The primary objective of this study was to investigate the incidence of LS in the Swedish population and to assess the correlation of comorbid conditions among patients diagnosed with LS compared to a control group without LS, with a special emphasis on its association with various types of cancer.

## 2. Methods

### 2.1. Study Design 

In this nationwide, retrospective cohort study, anonymized data encompassing all individuals diagnosed with LS (coded as L90.0 in the International Classification of Diseases, Tenth Revision, ICD-10) between 1 January 2001 and 1 January 2021 were obtained from the National Patient Register (NPR). The data contained patient information regarding sex, age, year of diagnosis, and comorbidity. The patients were only included the first time they received a diagnosis of LS. Once we identified our incident cases, we stratified them by sex and age. Age was categorized into 10-year bands ranging from 0–9, 10–19, …, 80–89 to 90+ years for all patients over 90 years. A matched control group was generated from the general population via Statistics Sweden, comprising 463,273 individuals, equating to three control subjects for every LS patient. Matching was based on age and sex at the time of LS diagnosis (Table 1). The control subjects had no documented prior diagnosis of LS. Data on the general Swedish population, categorized by sex, age, and year, were acquired from the Statistics Sweden database (www.scb.se, accessed on 9 April 2024). These data enabled the present study to associate incident cases to the general population. The entire Swedish population from 2001 to 2021 was considered at risk (*n* = 9,550,145) when this study estimated the age- and sex-specific incidence. LS incidence was estimated by dividing the mean number of cases diagnosed from 2001 to 2021 by the mean background population during the corresponding years. For comprehensive analysis, record linkage was enabled for both the LS and control cohorts with the NPR and the Swedish National Cancer Register, facilitated through everyone’s unique personal identification number (PIN). This linkage leverages the electronic integration present across these national databases. The selection of comorbid diagnoses for study was informed by a review of prior research, clinical insights, and patient historical data. The case group’s age was defined by their age at initial LS diagnosis (Table 1).

### 2.2. Swedish National Patient Register

Initiated in 1964 by the National Board of Health and Welfare, the Swedish NPR is a comprehensive source covering virtually all inpatient care within both the public and private sectors excluding primary care data. Patients diagnosed with LS by primary care and not by specialists are not recorded in the National Patient Register (NPR), and consequently were not included in this study. The register includes patient demographics (PIN, sex, age, county of residence), administrative, hospital identification, and medical data (diagnoses). From 1997, diagnoses within the NPR have been coded according to the ICD-10 revision. While inpatient coverage approaches 100%, outpatient coverage is reported at 87%. Mandatory outpatient reporting to the NPR was implemented in 2001, although primary care data remain outside its scope [34].

### 2.3. Swedish Cancer Register (SCR)

Established in 1958 by the Swedish National Board of Health and Welfare, the SCR provides exhaustive coverage of the Swedish population. Renowned for its accuracy, approximately 99% of cancer cases in the register are morphologically verified. The SCR captures data on tumor characteristics (location, histological type, diagnosis date and basis) along with follow-up information (dates of death, cause of death, and migration). Cancer case reporting is compulsory, with an impressive compliance rate of 96%. The register primarily relies on multiple reporting sources (clinicians, pathologists, cytologists) for case registration excluding cancer diagnoses derived solely from death certificates [35].

### 2.4. Ethical Considerations

This registry-based investigation employed data that had been rigorously pseudonymized in compliance with the General Data Protection Regulation (GDPR), negating the requirement for individual consent from the subjects involved. Ethical clearance for this study was secured from the Swedish Ethical Review Authority on 15 November 2021, under file number 2021-05590-01. 

### 2.5. Statistical Analysis

Data retrieved from the National Patient Register (NPR) and the Swedish Cancer Register (SCR) were compiled into an anonymized dataset, retaining only sex, age, and disease code, for analysis using IBM SPSS version 28.0. Age- and sex-specific incidence was calculated by dividing the number of cases in each age group by the total population in that age group per 100,000 inhabitants, with 85% confidence intervals (95%CI) computed using the Poisson normal approximation. Odds ratios (ORs) and adjusted ORs, along with 95% confidence intervals (95% CIs) and *p*-values for each diagnosis, were derived from binomial logistic regression. All ORs were adjusted for age, and in analyses of diagnoses applicable to both women and men, adjustments were also made for sex. A *p*-value of less than 0.05 was deemed indicative of statistical significance. Given the exploratory nature of this study, no adjustments for multiple comparisons were applied.

## 3. Results

**Figure 1:**
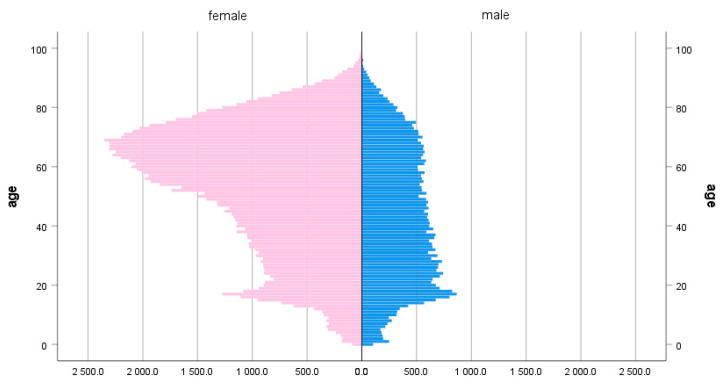
Population pyramid depicting the age dispersion of patients with lichen sclerosus, split by sex (females, n = 109,451, 70.9% and males, 44,973, 29.1%).

In this study, a total of 617,697 individuals were enrolled. Among them, 154,424 were identified as belonging to the case group and 463,273 were in the control group. In the case group, there were 44,973 (29.1%) males and 109,451 (70.9%) females, whereas the control group consisted of 134,919 (29.1%) males and 328,354 (70.9%) females. The median age at the time of diagnosis in the case group was 53 years, and the highest percentage of patients, 18.0%, was in the age group 60–69 (Table 1 and Figure 1).

**Figure 2:**
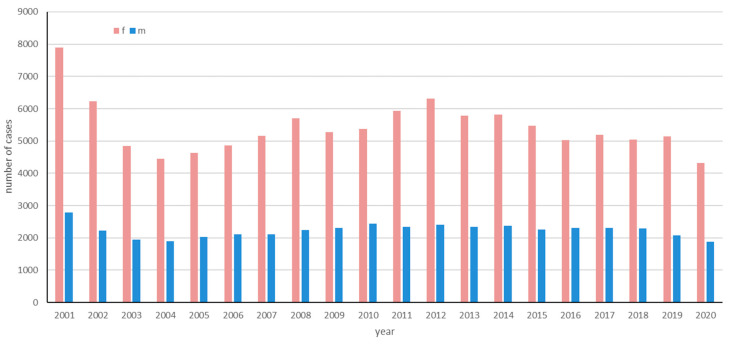
The number of incident cases of lichen sclerosus from 2001 to 2020 in Sweden based on data from the National Patient Register (NPR).

The incidence of LS was analyzed over a 20-year period (2001 to 2020). Figure 2 shows the number of incident cases for female and male patients between 2001 and 2020.

**Figure 3:**
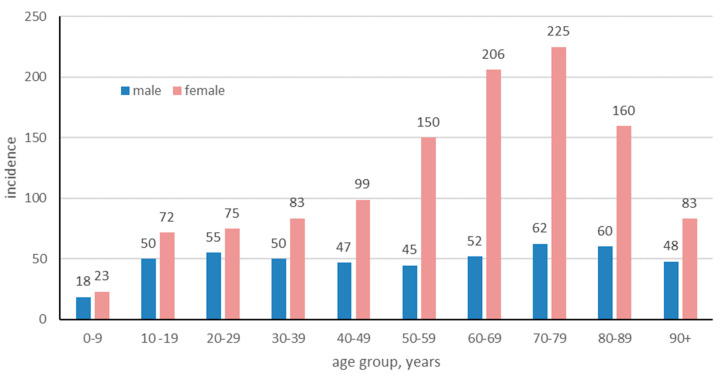
Age- and sex-specific incidence of lichen sclerosus per 100,000 in Sweden from 2001 to 2020.

The data included patients diagnosed at both inpatient and outpatient care units, but not primary care. Patients of all ages, diagnosed with LS between 2001 and 2020, were included in the study. The number of cases ranged from 4323 to 7901 per year for female patients and from 1875 to 2786 for male patients. The mean annual number of LS cases during those years was 7721.2; by dividing it by the mean background population during that period (n = 9,550,145), the incidence was estimated. The mean annual incidence of LS in Sweden 2001 to 2020 was 7721.2/9,550,145 = 80.9 cases/100,000 persons. When disaggregated by gender, the mean annual incidence for females was significantly higher, recorded at 114.4 new cases per 100,000 persons/year, compared to males, who presented a lower incidence rate of 47.2 new cases per 100,000 persons/year. The mean annual incidence peaked for male and female patients in the 70–79 age group with 62.4/100,000 for males, and with 224.8/100,000 (Figure 3). In females of fertile age (20–39 years old), the incidence of LS was notably prominent, comprising approximately 17.43% of the female LS patient population in the study. The incidence for female patients were significantly higher in all age groups except for those 0–9 and over 90 years of age (Table 2).

Our data revealed that individuals with LS exhibited a significant increased association for various malignant and premalignant conditions studied. This included an increased odds ratio (OR) risk for vulvar cancer (OR = 8.3; 95% confidence interval [CI] = 7.5–9.0), penile cancer (OR = 8.9; 95% CI = 7.3–11.0), prostate cancer (OR = 1.2; 95% CI = 1.1–1.2), testicular cancer (OR = 1.4; 95% CI = 1.1–1.7), bladder cancer (OR = 1.1; 95% CI = 1.1–1.2), breast cancer (OR = 1.4; 95% CI = 1.3–1.4), leukoplakia of the vulva (OR = 253.5; 95% CI = 221.9–289.6), and leukoplakia of the penis (OR = 5.1; 95% CI = 4.9–5.4), as detailed in Table 3. Additionally, the analysis identified a significant association between LS and various diseases including alopecia areata, lichen planus, morphea, vitiligo, systemic lupus erythematosus, systemic sclerosis, pemphigus, pemphigoid and other bullous dermatoses, psoriasis, atopic dermatitis, keratitis, thyroiditis, hyperthyroidism, ulcerative colitis, Crohn’s disease, diabetes mellitus type 1, non-seropositive rheumatoid arthritis, and multiple sclerosis, as outlined in Table 3.

## 4. Discussion 

In this study, we observed the anticipated bimodal age distribution of LS primarily within the female cohort, consistent with prior research [22,36]. Interestingly, this pattern did not manifest in the male population, where a single peak in incidence emerged prominently around 20 years of age. The absence of a second peak in older males raises speculation. One plausible explanation for this difference could be related to the presentation of LS in older men, who might only experience mild symptoms and consequently might not seek medical attention, potentially leading to underdiagnosis in this demographic. Alternatively, the lack of age-related fluctuations in the serum estrogen levels in men, as opposed to women, may account for a more uniform distribution of LS across ages in the male population [37,38,39]. Research focusing on postmenopausal women has demonstrated that decreased estrogen levels contribute to various skin changes including dryness, thinning, and delayed healing, factors that could exacerbate the visibility and diagnosis rates of LS in older females [37].

Consistent with prior research including one of our own investigations, we identified a significant correlation between LS and both leukoplakia of the vulva and vulvar cancer [24]. Although there is some evidence from case reports and our research findings, larger studies establishing a link between LS and penile cancer remain scarce [36]. Our current analysis revealed an increased prevalence of penile leukoplakia and penile malignancies among LS patients. A noteworthy limitation of our study is the potential overlap in diagnosis between patients identified with LS and those with leukoplakia, which could potentially amplify the association between LS and leukoplakia. This aspect calls for a careful interpretation of the connection between these conditions as reported in our study. Building on our findings, we advocate for a critical reassessment of the existing national clinical guidelines pertaining to LS, particularly concerning the diagnosis, treatment, screening, and longitudinal monitoring of male patients affected by this condition. The evidence presented in this study, emphasizing the significant association of LS with conditions such as leukoplakia and penile cancer, underscores the imperative need for an updated approach that encompasses these important correlations. Such a revision should aim to incorporate the latest research findings and epidemiological data to ensure that clinical practices are aligned with the evolving understanding of LS and its associated risks. Moreover, this update should consider implementing comprehensive screening strategies and follow-up protocols specifically tailored to address the unique presentation of LS in males, thereby enhancing patient outcomes through early detection, precise diagnosis, and optimized treatment pathways.

Research exploring the connection between LS and cancer risks beyond vulvar malignancies is limited. Our findings indicate a significant association between LS and breast cancer, presenting a divergence from earlier studies [40]. The reasons behind these differing outcomes may relate to genetic or geographical variations within the studied populations. To resolve these discrepancies and gain a clearer understanding of the link between LS and breast cancer, further research is warranted. Future investigations should entail comprehensive, nationwide register studies to explore this association more thoroughly.

Additionally, our analysis has uncovered significant associations between LS and prostate, testicular, and bladder cancers, areas previously unexplored outside of our research group’s work on prostate cancer. The inclusion of these cancers was prompted by clinical observations and patient histories indicating a potential link between penile LS and these malignancies. This insight underscores the necessity for more extensive research to validate these preliminary associations and to understand the mechanisms underlying the relationship between LS and these specific cancers.

Consistent with prior research, our study found no notable association between seropositive rheumatoid arthritis (RA) and LS in comparison to the control group. However, an elevated odds ratio (OR) for non-seropositive RA among LS patients was observed. Notably, this study identified a significant link between systemic lupus erythematosus (SLE) and LS, extending beyond the limited case reports previously documented. This association has been corroborated by recent findings from a Finnish case–control study that reported an increased risk of SLE among individuals with LS [11,31,32,41,42,43].

Additionally, our research revealed a significant increased OR for type 1 diabetes mellitus (DM1) in LS patients. In line with this, a study by Virgilli et al. in Italy highlighted that LS patients exhibited a higher prevalence of overweight and obesity compared to the general Italian population [44,45]. Similarly, American research reinforcing earlier data demonstrated that autoimmune thyroid disease was more prevalent among LS patients, with significant increases in the odds of thyroiditis, autoimmune thyroiditis, hypothyroidism, and hyperthyroidism [11,42,43,46,47]. While one study did not establish a significant connection between thyroiditis and LS, particularly in women [42], our findings indicate an increased risk for both hyperthyroidism and thyroiditis in LS patients, aligning with the earlier mentioned studies.

Moreover, our study’s results align with previous reports regarding the frequent co-occurrence of lichen planus with LS lesions, thereby strengthening the established association between these conditions. This observation further underscores the intricate interplay between LS and a diverse array of autoimmune and endocrine disorders. It emphasizes the necessity for a comprehensive, holistic approach to the management and surveillance of individuals diagnosed with LS.

This study reaffirms the established associations between LS and a spectrum of autoimmune diseases including alopecia areata, ulcerative colitis [48], and vitiligo [29,30], thereby reinforcing the documented linkage of LS with these conditions. Notably, our findings also elucidate a novel association between LS and Crohn’s disease, a connection not previously documented outside of investigations conducted by our research group [36].

Additionally, the co-occurrence of vasculitis with LS, as identified in previous research, may be attributable to the shared presence of HLA-DR bearing keratinocytes in both conditions, accompanied by a lymphocytic infiltrate rich in activated T-cells [49]. This observation highlights the potential shared pathogenetic mechanisms between LS and vasculitis, emphasizing the intricate immune-mediated interrelations underlying LS and its comorbid conditions.

Elevated odds ratios for morphea, systemic sclerosis, and SLE among patients with LS have been observed in our study, suggesting a notable association between these conditions. Research from the USA highlighted an increased prevalence of LS in postmenopausal women diagnosed with morphea, with a significant proportion of those exhibiting genital involvement of morphea also presenting clinical and histopathological characteristics indicative of LS. Specifically, 59.2% of patients with genital morphea demonstrated concurrent extragenital LS and overlapping morphea plaques, underscoring the frequent co-occurrence of LS and morphea, though the nature of their relationship remains a topic of ongoing debate [33]. Intriguingly, a study from France identified a significant correlation between genital LS and the limited cutaneous form of systemic sclerosis pointing toward a subset-specific association within the spectrum of systemic sclerosis. Nevertheless, this investigation did not establish a broader association between LS and systemic sclerosis across all its subsets [50,51]. Contrary to these findings, our current analysis revealed a positive association between LS and systemic sclerosis, inclusive of all its subsets, thus prompting further investigative efforts to elucidate the underlying dynamics of their relationship.

### Strengths and Limitations

There is potential bias in our cohort, as some LS patients may be missing from our data. This could be due to various factors such as mild symptoms leading to underdiagnosis, misdiagnosis, or patients not seeking medical assistance. Additionally, the diagnostic criteria for LS have evolved over time, possibly becoming more precise. It is important to note that LS diagnosis is primarily based on clinical assessment and may not always be confirmed by histopathology. Unfortunately, we lacked data regarding whether LS diagnoses in the cases included in our study were confirmed by biopsy.

A potential limitation of this study is rooted in the data sourcing strategy, particularly the reliance on the Swedish National Patient Register, which does not include diagnoses made in primary care settings. This registry limitation means our analysis is confined to cases identified by specialists only, inherently omitting a segment of the population potentially diagnosed with LS in a primary care context. The exclusion of primary care diagnoses could lead to an underrepresentation of LS incidence rates, particularly for cases with milder symptomatology that do not necessitate specialist referral. Conversely, this methodological constraint may contribute to a heightened diagnostic specificity within our dataset, as it presumably lowers the prevalence of false-positive LS diagnoses. Acknowledging this limitation is crucial for interpreting our findings, as it highlights the potential for both an underestimation of true LS prevalence and an artificially enhanced accuracy in the identification of confirmed cases.

In Sweden, circumcision is not a standard practice for newborns or conducted upon request but is reserved for severe cases of phimosis. It is noteworthy that the foreskin removed during these procedures often does not undergo histopathological evaluation, potentially overlooking LS diagnoses. Despite this practice, it is crucial to acknowledge the absence of specific data regarding the frequency of undiagnosed LS in such cases. Therefore, while it may have some bearing on the study’s findings, the influence of this practice on the overall results is minimal.

Moreover, the process of manually inputting ICD-10 codes into patient records can occasionally lead to errors, with incorrect codes being assigned. Despite this, the ICD-10 framework is a globally accepted diagnostic coding system, and healthcare professionals undergo training to minimize such inaccuracies. As a result, the likelihood of these errors is low, and their impact on the study’s findings is expected to be minimal.

This investigation has identified various associations between LS and a range of other diagnoses, aligning with and diverging from previous research findings. This underscores the necessity for further exploration into LS, emphasizing the complexity of its relationships with other health conditions and the potential for new insights into its etiology and interactions.

## 5. Conclusions

This study corroborates the known risk of vulvar and penile cancers associated with LS and introduces pivotal insights into the links of LS with prostate, breast, testicular, and bladder cancers, among other conditions. These findings underscore the necessity for expansive national research to validate these associations comprehensively. Such research is critical for refining our understanding of LS, potentially leading to revised treatment protocols, cancer screening, and follow-up procedures. Additionally, the study highlights the need for further investigation into the etiology and pathophysiology of LS, particularly its malignant potential, which could pave the way for novel therapeutic strategies. Accordingly, our results call for an urgent update of the national clinical guidelines for LS, especially regarding the management of male patients. By integrating the latest scientific discoveries and epidemiological data, we can enhance clinical practices to reflect the nuanced relationships between LS and its associated risks, ultimately improving patient outcomes through targeted screening and personalized care plans.

## Figures and Tables

**Table 1:** Demographic data for patients diagnosed with lichen sclerosus and the control cohort.

	Cases			Controls		
	*n* = 154,424	F = 109,451 (70.9%)	M = 44,973 (29.1%)	*n* = 463,273	F = 328,354 (70.9%)	M = 134,919 (29.1%)
Median age (min–max)	53 (0–103)	57 (0–103)	42 (0–100)	53 (0–103)	57 (0–103)	42 (0–100)
Age group	*n* (%)	*n*		*n* (%)	*n*	
0–9	4503 (2.9)	2439	2064	13,509 (2.9)	7317	6192
10–19	13,729 (8.9)	7865	5864	41,187 (8.9)	23,595	17,592
20–29	15,690 (10.2)	8825	6865	47,070 (10.2)	26,475	20,595
30–39	16,713 (10.8)	10,259	6454	50,139 (10.8)	30,777	19,362
40–49	18,338 (11.9)	12,318	6020	55,014 (11.9)	36,954	18,060
50–59	23,558 (15.3)	18,078	5480	70,674 (15.3)	54,234	16,440
60–69	27,818 (18.0)	22,288	5530	83,454 (18.0)	66,864	16,590
70–79	22,894 (14.8)	18,395	4499	68,682 (14.8)	55,185	13,497
80–89	9935 (6.4)	7969	1966	29,805 (6.4)	23,907	5898
90+	1246 (0.8)	1015	231	3739 (0.8)	3046	693

*n* = number, M = male, F = fem.

**Table 2:** The average annual incidence of lichen sclerosus per 100,000 in Sweden, 2001–2020, stratified by age group and sex.

Age Group	Incidence Females	95% CI *	Incidence Males	95% CI
0–9	23.0	18.9–27.1	18.4	14.9–22.0
10–19	71.5	**64.5–78.6**	50.2	**44.5–56.0**
20–29	74.6	**67.7–81.6**	55.1	**49.3–60.9**
30–39	83.4	**76.2–90.6**	50.3	**44.8–55.7**
40–49	98.7	**90.9–106.5**	46.6	**41.4–51.9**
50–59	149.8	**140.0–159.5**	44.5	**39.3–49.8**
60–69	206.0	**193.9–218.1**	51.8	**45.7–57.9**
70–79	224.8	**210.2–239.3**	62.4	**54.2–70.5**
80–89	159.6	**143.9–175.3**	60.3	**48.4–72.2**
90+	83.0	60.2–105.9	47.7	20.2–75.3

* Confidence interval calculated according to Poisson normal approximation. Significant values shown in **bold**.

**Table 3:** Number, frequency, and odds ratio for comorbid conditions of patients with lichen sclerosus compared to controls.

Comorbidity	ICD-10 Code	Lichen Sclerosus *n* (%)	Controls *n* (%)	OR (95% CI)	adj OR ^c^ (95%CI)	Significance Level ^d^
Neoplasms						
Breast cancer ^a^	C50	7225 (6.6)	16,258 (4.9)	1.4 (1.3–1.4)	1.3 (1.3–1.4)	***
Vulvar cancer ^a^	C51	1680 (1.5)	622 (0.2)	8.2 (7.5–9.0)	8.3 (7.5–9.1)	***
Penile cancer ^b^	C60	367 (0.8)	124 (0.1)	8.9 (7.3–11.0)	9.0 (7.3–11.0)	***
Prostate cancer ^b^	C61	2342 (5.2)	6152 (4.6)	1.2 (1.1–1.2)	1.2 (1.1–1.2)	***
Testicular cancer ^b^	C62	130 (0.3)	284 (0.2)	1.4 (1.1–1.7)	1.4 (1.1–1.7)	**
Bladder cancer	C67	1057 (0.7)	2811 (0.6)	1.1 (1.1–1.2)	1.1 (1.1–1.2)	***
Urethral cancer	C68	46 (0.03)	104 (0.02)	1.3 (0.9–1.9)	1.3 (0.9–1.9)	ns
Endocrine, nutritional and metabolic diseases						
Hyperthyroidism	E05	2938 (1.9)	7395 (1.6)	1.2 (1.1–1.3)	1.2 (1.2–1.3)	***
Thyroiditis	E06	1146 (0.7)	2169 (0.5)	1.6 (1.5–1.7)	1.6 (1.5–1.7)	***
Type 1 diabetes mellitus	E10	3909 (2.5)	9395 (2.0)	1.3 (1.2–1.3)	1.3 (1.2–1.3)	***
Obesity	E66	9157 (5.9)	18,414 (4.0)	1.5 (1.5–1.6)	1.5 (1.5–1.6)	***
Mental and behavioral disorders						
Mental and behavioral disorders due to use of alcohol	F10	5117 (3.3)	13,173 (2.8)	1.2 (1.1–1.2)	1.2 (1.1–1.2)	***
Diseases of the nervous system						
Multiple sclerosis	G35	608 (0.4)	1769 (0.4)	1.0 (0.9–1.1)	1.0 (0.9–1.1)	ns
Inflammatory polyneuropathy/Guillain Barre syndrome	G61	195 (0.13)	403 (0.08)	1.5 (1.2–1.7)	1.5 (1.2–1.7)	***
Myasthenia gravis and other myoneural disorders	G70	147 (0.1)	309 (0.06)	1.4 (1.2–1.7)	1.4 (1.2–1.7)	***
Diseases of the eye						
Keratitis	H16	5670 (3.7)	11,961 (2.6)	1.4 (1.4–1.5)	1.4 (1.4–1.5)	***
Iridocyclitis	H20	2949 (1.9)	6985 (1.5)	1.3 (1.2–1.3)	1.3 (1.2–1.3)	***
Diseases of the digestive system						
Crohn’s disease	K50	1686 (1.1)	2812 (0.6)	1.8 (1.7–1.9)	1.8 (1.7–1.9)	***
Ulcerative colitis	K51	2115 (1.4)	4272 (0.9)	1.5 (1.4–1.6)	1.5 (1.4–1.6)	***
Diseases of the skin						
Pemphigus	L10	220 (0.14)	398 (0.08)	1.7 (1.4–2.0)	1.7 (1.4–2.0)	***
Pemphigoid	L12	291 (0.2)	500 (0.1)	1.8 (1.5–2.0)	1.8 (1.5–2.0)	***
Other bullous disorders	L13	225 (0.15)	360 (0.07)	1.9 (1.6–2.2)	1.9 (1.6–2.2)	***
Atopic dermatitis	L20	4909 (3.2)	8191 (1.8)	1.8 (1.8–1.9)	1.9 (1.8–1.9)	***
Psoriasis	L40	6588 (4.3)	12,224 (2.6)	1.6 (1.6–1.7)	1.7 (1.6–1.7)	***
Lichen planus	L43	4679 (3.0)	2566 (0.6)	5.6 (5.4–5.9)	5.6 (5.4–5.9)	***
Alopecia areata	L63	909 (0.6)	1209 (0.3)	2.3 (2.1–2.5)	2.3 (2.1–2.5)	***
Vitiligo	L80	1217 (0.8)	936 (0.2)	3.9 (3.6–4.3)	3.9 (3.6–4.3)	***
Localized scleroderma (morphea)	L94	855 (0.6)	418 (0.1)	6.2 (5.5–6.9)	6.2 (5.5–6.9)	***
Vasculitis limited to skin	L95	455 (0.3)	593 (0.1)	2.3 (2.0–2.6)	2.3 (2.0–2.6)	***
Diseases of the musculoskeletal system and connective tissue						
Seropositive rheumatoid arthritis	M05	1977 (1.3)	5769 (1.2)	1.0 (0.9–1.1)	1.0 (0.9–1.1)	ns
Other rheumatoid arthritis	M06	2283 (1.5)	5987 (1.3)	1.2 (1.1–1.2)	1.2 (1.1–1.2)	***
Polyarteritis nodosa and related conditions	M30	91 (0.06)	172 (0.03)	1.6 (1.2–2.1)	1.6 (1.2–2.1)	***
Other necrotizing vasculopathies	M31	1172 (0.8)	2481 (0.5)	1.4 (1.3–1.5)	1.4 (1.3–1.5)	***
Systemic lupus erythematosus	M32	465 (0.3)	914 (0.2)	1.5 (1.4–1.7)	1.5 (1.4–1.7)	***
Systemic sclerosis	M34	246 (0.2)	324 (0.1)	2.3 (1.9–2.7)	2.3 (1.9–2.7)	***
Diseases of the genitourinary system						
Leucoplakia of penis ^b^	N48.0	4878 (3.2)	3126 (0.7)	5.1 (4.9–5.4)	5.2 (4.9–5.4)	***
Leucoplakia of Vulva ^a^	N90.4	15,965 (10.3)	221 (0.05)	253.5 (221.9–289.6)	262.9 (230.2–300.3)	***
Factors influencing health status						
Tobacco use	Z72.0	3012 (2.0)	7954 (1.7)	1.1 (1.1–1.2)	1.1 (1.1–1.2)	***

adj OR = adjusted odds ratio, ^a^ Only females were included in the regression analysis, ^b^ Only males were included in the regression analysis, ^c^ Odds ratio adjusted for age group and sex, if both sexes were included in the regression analysis, ^d^ Significance level of adjusted odds ratio (** *p* < 0.01; *** *p* < 0.001; ns *p* > 0.05).

## Data Availability

Data and materials can be assessed by contacting one of the authors.
